# Heavy ion beam-induced variation in salt tolerance among *Leymus chinensis* genotypes during seed germination

**DOI:** 10.7717/peerj.20510

**Published:** 2025-12-17

**Authors:** Shiman Chen, Yaxiao Li, Dandan Zhao, Mengdan Sun, Wen wen Qi, Ming xue Shi, Shaoyang Li, Hongyuan Ma

**Affiliations:** 1 University of Chinese Academy of Sciences, Beijing, China; 2 Northeast Institute of Geography and Agroecology, Chinese Academy of Sciences, Jilin, Changchun, China; 3 Harbin Normal University, Heilongjiang, China; 4 Key Laboratory of Vegetation Ecology of the Ministry of Education, Jilin, Changchun, China

**Keywords:** Heavy ion beam irradiation, Perennial forage grass, Germination, Saline stress, Genotype specificity

## Abstract

Heavy ion beam irradiation (HIBI) is an efficient mutagenic tool characterized by high linear energy transfer and its capacity to induce heritable genetic variation. However, its application in perennial forage species remains limited, especially in terms of genotype-specific responses to radiation and salt tolerance. In this study, nine *Leymus chinensis* genotypes were irradiated with carbon ion beams at doses of 40, 50, and 80 Gy, with 0 Gy as the control. Germination responses were then evaluated under non-saline (distilled water) and saline (100 mmol·L^−1^ NaCl) conditions to assess the effects of irradiation and genotype-specific salt tolerance. Both radiation dose and salt stress significantly affected early seedling development, showing pronounced genotype-dependent variation. Under 80 Gy combined with salt stress, emergence percentages of sensitive genotypes (*e.g*., G2, G4) dropped below 10%, whereas tolerant genotypes (*e.g*., G3, G9) exhibited a 19.3–65.1% increase under high-dose conditions. The half-lethal dose (LD_50_), calculated based on emergence data, ranged from 29.6 Gy (G4) to over 80 Gy (G1), allowing classification into three salt tolerance levels: high, medium, and low. Principal component analysis (PCA) supported the trait-based classification and revealed synergistic variation patterns under combined stress conditions. These findings highlight the potential of HIBI to induce beneficial traits for salt tolerance in *L. chinensis*, providing a theoretical foundation for precision breeding and ecological restoration in saline grasslands.

## Introduction

Soil salinization is a major challenge facing global ecology and agricultural production today. According to a December 2024 report from the Food and Agriculture Organization (FAO), over 1.381 billion hectares of land (approximately 10.7% of the world’s total) are affected by salinity. Salinity stress not only leads to deterioration of soil physical and chemical properties, imbalance of microbial communities, and degradation of the rhizosphere environment, but also severely disrupts grassland vegetation structure and ecological functions, resulting in reduced grassland productivity and weakened ecosystem stability (Kumar et al., 2017; Ramadan et al., 2023). In grassland systems, these stressors significantly reduce biomass and lead to widespread degradation, particularly through the loss of high-quality perennial forage species.

Perennial forage grasses are vital to the productivity and resilience of grassland ecosystems. In addition to sustaining primary productivity, they contribute significantly to carbon sequestration, soil stabilization, water retention, and the formation of ecological buffers (Halli et al., 2022). However, establishing these species in saline-alkali soils remains a major challenge due to poor germination and low seedling establishment under saline environments. Enhancing the salt tolerance and early developmental performance of forage germplasm is therefore essential for effective grassland restoration and the sustainable development of forage-based livestock systems (Chen et al., 2014; Wang et al., 2024).

Heavy ion beam irradiation (HIBI) is characterized by high linear energy transfer (LET), elevated relative biological effectiveness (RBE), precise spatial energy deposition, and limited post-damage DNA repair (Guo et al., 2024). These unique physical attributes make HIBI a powerful tool for inducing targeted genetic variation in plant breeding programs (Du et al., 2021; Ren et al., 2025). Compared with traditional γ-rays, HIBI-induced DNA double-strand breaks (DSBs) are more likely to produce heritable mutations (mutation frequency increased by 4–6 times) and affect the expression regulatory network of stress response genes (such as SOG5 and P5CS) by regulating transposon activation (methylation level changes by 15–22%) and chromatin remodeling (Hagiwara et al., 2019; Ito, 2022).

Given its ability to induce diverse mutations, HIBI has been widely adopted for germplasm enhancement in crops such as *Arabidopsis thaliana* (Li et al., 2020; Wang et al., 2018a, 2018b; Kazama et al., 2011) and rice (Ren et al., 2023), supporting its use in germplasm innovation (Miyagi et al., 2018). Functional studies and mutation spectrum analyses further highlight its potential compared to traditional methods like γ-ray or X-ray irradiation (Du et al., 2022; Li et al., 2019). Previous studies have shown that low-dose carbon ion beam radiation promotes the growth of Arabidopsis seedlings by increasing ROS levels and inducing antioxidant system activity (Wang et al., 2018a), ^60^Co-γ ray irradiation can significantly increase the number of tillers, plant height, and fresh weight of ryegrass (Ramadan et al., 2023). However, the application of HIBI in perennial forage species such as *L. chinensis* remains extremely limited, and comprehensive studies on radiation-induced mutagenesis and salt tolerance screening in this species are still lacking.

*L. chinensis* is a dominant perennial forage species across the Eurasian steppe, known for its strong tolerance to cold, drought, and salinity, as well as high nutritional value and palatability (Li et al., 2023; Zhao et al., 2016). Considerable phenotypic variation exists among its genotypes, particularly in traits such as seed germination, seedling growth, and leaf coloration, which are largely shaped by underlying genetic differences (Ahmed & Hou, 2022; Gong et al., 2007). Such genotype-specific traits not only determine the baseline salt tolerance of different accessions but also influence their mutagenic responses to irradiation. As heavy ion beam irradiation can trigger a wide spectrum of genetic and epigenetic changes, it is reasonable to expect that individual genotypes will vary in both sensitivity to irradiation dose and in their ability to cope with salt stress (Shi et al., 2025). This implies potential three-way interactions (genotype × irradiation dose × salinity) that remain unexplored in perennial forage species. It plays a vital role in maintaining grassland productivity and ecological stability. However, natural populations have suffered widespread degradation due to long-term overgrazing, climate change, and increasing soil salinization. The limited salt tolerance of current cultivars during germination severely hampers their artificial establishment in saline-alkali soils (Li, Huang & Li, 2021). Given that germination is the most sensitive stage in the plant life cycle, improving salt tolerance at this phase is essential for enhancing seedling establishment and population renewal (Tarchoun et al., 2024; Yan et al., 2022). Therefore, the development of salt-tolerant germplasm at the germination stage is a key focus in breeding programs aimed at improving the resilience and sustainability of grassland forage systems (Fu et al., 2024; Guo et al., 2022; Li et al., 2021; Ramadan et al., 2023; Wang et al., 2024).

In this context, this study investigates the phenotypic responses of *L. chinensis* to HIBI under salt stress, with a focus on seed germination and early seedling development across nine genotypes. The research specifically aims to: (1) evaluate differences in germination and early growth traits under varying irradiation doses and salt stress conditions; (2) compare genotype-specific phenotypic responses to combined radiation and salinity stress; and (3) explore the potential application of HIBI as a mutagenic strategy for improving salt tolerance in perennial forage grasses. By explicitly testing for genotype × dose × salinity interactions, this study provides new insight into how genetic background shapes the outcomes of mutagenic and environmental stress factors. By characterizing dose-dependent and genotype-dependent variation in key traits, this study provides a theoretical basis and technical reference for the development of salt-tolerant *L. chinensis* germplasm and the ecological restoration of saline grasslands.

## Materials and Methods

### Materials

Nine genotypes of *Leymus chinensis* (G1–G9) were used in this study. The detailed information on their origin, type, and key morphological traits is summarized in Table S1. These genotypes include cultivars, breeding lines, and wild accessions that represent diverse habitats and ecotypes in Northeast China. All germplasm resources were maintained at the experimental fields of the Northeast Institute of Geography and Agroecology, Chinese Academy of Sciences (CAS, Jilin, China). Mature and plump seeds of uniform size were carefully selected, affixed to transparent adhesive tape, and placed in 3-cm-diameter Petri dishes for irradiation treatment.

### Heavy ion beam irradiation treatment

This experiment was conducted at the biological terminal of the Heavy Ion Research Facility (HIRFL) in the National Key Laboratory of Heavy Ion Accelerators at the Institute of Modern Physics, Chinese Academy of Sciences. The carbon ion beam used was ^12^C^6+^ with an initial energy of 80 MeV/u, which was irradiated onto the seed surface through a stainless-steel window, Mylar film, and air. All irradiation experiments were conducted under atmospheric conditions and at room temperature, with sample replacement and data acquisition controlled by a computer. The dose percentage was 40 Gy/min, with irradiation doses set at 40, 50, and 80 Gy. Seeds with an irradiation dose of 0 Gy served as the control group.

### Germination test

Following irradiation, seeds were surface-sterilized in 0.1% HgCl_2_ for 10 min, thoroughly rinsed with distilled water, and air-dried on sterile filter paper. For each treatment, 20 seeds per genotype were placed on 9-cm Petri dishes containing 0.7% agar medium. Two types of media were used: (1) distilled water (control) and (2) 100 mM NaCl (salt stress). The media were sterilized in an autoclave (Model SQ810C, YAMATO, Japan) at 120 °C for 15 min before use. The experimental design included 72 treatment combinations (9 genotypes × 4 doses × 2 salinity levels), each with four replicates, for a total of 288 dishes.

Plates were incubated in an illuminated growth chamber (Model PGX-450C, Saifu, China) under a temperature regime of 28 °C (12 h light) and 16 °C (12 h dark). Germinated seeds were counted daily, and the test was concluded when no new germination was observed for three consecutive days (up to day 28). At the end of the germination period, primary root length (from root tip to root–hypocotyl junction) and shoot height (from stem base to the tip of the longest leaf) were measured with a vernier caliper. Additional morphological traits, including root number and leaf number, were also recorded.

### Trait definitions and LD_50_ estimation

Germination percentage and emergence percentage were calculated using the following formulas:

${\mathrm{Germination}}\;{\mathrm{percentage}}\;(\% ) = {n \over N} \times 100\% ,{\mathrm{Emergence}}\;{\mathrm{percentage}}\;(\% ) = {{{{\mathrm{n}}^{'}}} \over N} \times 100\% $

where n is the number of germinated seeds (radicle emergence), n' is the number of emerged seedlings (visible coleoptile or shoot), and N is the total number of seeds per dish.

The LD_50_ lethal dose 50% (LD_50_) (Shu & Nakagawa, 2011) was calculated for each genotype based on the dose–response relationship of emergence percentage. A simple linear regression model was fitted for each genotype:

$${\rm Y = aX + b}$$

where Y is the emergence percentage (%), X is the irradiation dose (Gy), and a and b are regression coefficients. The LD_50_ was defined as the irradiation dose corresponding to 50% of the emergence percentage observed in the control group (0 Gy), calculated as:

${\rm L}{{\rm D}_{{\rm 50}}}=\displaystyle{{{\rm 0}{\rm .5}{{\rm Y}_{\rm 0}}-{\rm b}} \over {\rm a}}$

where Y_0_ is the control (0 Gy) emergence percentage of the given genotype.

### Statistical analysis

Generalized linear models (GLMs) based on the binomial distribution were used to analyze the effects of heavy ion beam irradiation and NaCl treatment on the germination percentage and survival percentage of *L. chinensis* seeds of different genotypes. General linear models (LMs) were used to analyze the effects of heavy ion beam irradiation and NaCl treatment on the root length and shoot length of *L. chinensis* seeds of different genotypes. *Post hoc* tests were performed using Tukey’s test, with a significance level of 0.05.

A linear regression model was used to estimate LD_50_ for each genotype. Principal component analysis (PCA) based on germination, emergence, root length, and shoot length was conducted to assess trait variation and genotype responses. All statistical analyses and visualizations were conducted in R 4.2.2.

## Result

### Seed germination of *Leymus chinensis* under HIBI and salinity stress

Radiation dose and salt stress significantly affected the germination percentage of *L. chinensis*, with strong genotype-dependent differences (*p* < 0.001). A significant three-way interaction among genotype, radiation dose, and salinity was detected (*p* < 0.001), indicating genotype-specific combined effects (Table 1).

**Table 1:** Statistical effects of genotype (G), salinity (S), radiation dose (R), and their interactions on seed germination percentage (GP), emergence percentage (EP), root length (RL), and shoot length (SL) of *L. chinensis*. Germination- and emergence-related traits were analyzed using generalized linear models (GLMs, binomial distribution), while root and shoot traits were analyzed using linear models (LMs). Significant *p*-values are shown in bold (*p* < 0.05).

Factors	GP			EP			RL			SL		
	Df	$\chi^2$	P	Df	$\chi^2$	P	Df	F	P	Df	F	P
G	8	599.34	**<0.001**	8	649.67	**<0.001**	8	7.80	**<0.001**	8	10.54	**<0.001**
S	1	13.24	**<0.001**	1	2.94	0.086	1	0.30	0.585	1	1.87	0.172
R	3	10.77	0.013	3	244.10	**<0.001**	3	205.35	**<0.001**	3	131.60	**<0.001**
G × S	8	32.85	**<0.001**	8	16.10	0.041	7	2.43	0.018	7	1.51	0.160
G × R	24	144.57	**<0.001**	24	99.31	**<0.001**	24	1.38	0.106	24	3.93	**<0.001**
S × R	3	2.75	0.432	3	6.25	0.100	3	3.51	0.015	3	0.62	0.599
G × S × R	24	55.81	**<0.001**	24	70.26	**<0.001**	24	1.68	0.030	24	2.64	**<0.001**

**Note:**

Df, Degrees of freedom; $\chi^2$, chi-square statistic; F, F-statistic; GP, germination percentage; EP, emergence percentage; RL, root length; SL, shoot length.

**Figure 1:**
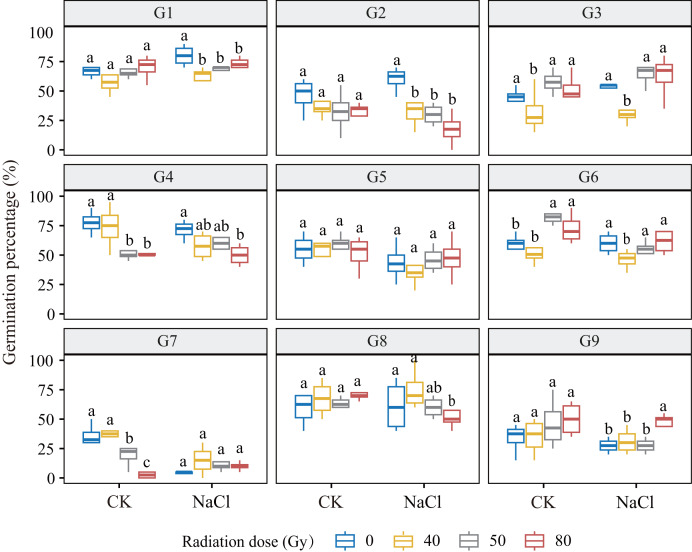
Germination percentage of nine *L. chinensis* genotypes (G1–G9) under different carbon ion beam irradiation doses (0, 40, 50, 80 Gy) and salinity treatments. Boxplots show the distribution of germination percentages (%) for each genotype across treatments. Different lowercase letters indicate significant differences among radiation dose groups within each salinity condition.

Under non-saline conditions, germination responses to increasing radiation doses varied across genotypes (Fig. 1). G1, G3, G6, and G9 showed a dose-dependent increase in germination, with G6 exhibiting the greatest stimulation at low doses (*p* < 0.001). In contrast, G2, G4, and G7 exhibited a dose-dependent decline, with G7 being most sensitive. G5 and G8 showed no significant variation, suggesting radiation tolerance. Under salt stress (100 mM NaCl), no significant dose × salinity interaction was observed overall. However, genotype-specific trends remained evident: G2, G4, and G8 showed decreased germination with increasing dose, while G3 and G9 showed significant increases. Notably, G3 germination increased by 19.3% at 50 Gy compared to 40 Gy (*p* ≤ 0.001), and G9 increased by 65.1% at 80 Gy compared to the control, indicating high salt-radiation adaptability.

### Seedling emergence percentage and radiation sensitivity classification

A significant three-way interaction among genotype, radiation dose, and salt stress was observed for seedling emergence percentage (*p* < 0.001), with a highly significant genotype × dose interaction also detected (*p* < 0.001; Table 1). This highlights the influence of genetic background on emergence performance under combined stress conditions.

**Figure 2:**
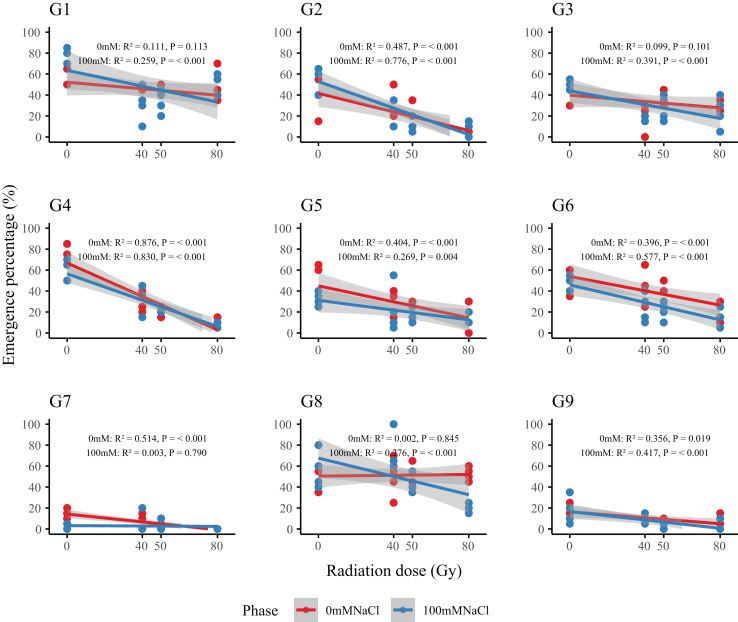
Dose–response relationships between radiation dose and emergence percentage (%) in nine *L. chinensis* genotypes (G1–G9) under two salinity treatments. Each panel represents a linear regression model for a single genotype under control (0 mM NaCl, red line) and salt-stressed conditions (100 mM NaCl, blue line). Shaded areas indicate 95% confidence intervals. Coefficients of determination (R^2^) and *p*-values are shown for each fitted model.

Dose–response analysis revealed a general decline in emergence with increasing radiation dose (Fig 2). Genotypes G2 and G4 were particularly sensitive: at 80 Gy, emergence in G2 dropped from 41.3–57.5% (control) to 6.3–8.8%, while G4 decreased from 58.8–73.8% to 8.8–11.3%. In contrast, G1, G3, and G6 maintained stable emergence across all doses, indicating strong radiation tolerance. Regression-derived LD_50_ values (Table 2) allowed classification of genotypes into three resistance groups: high (G1, G3, G6; LD_50_ > 80 Gy), moderate (G5, G7, G9; LD_50_= 50–80 Gy), and low (G2, G4; LD_50_ < 50 Gy). This classification provides a basis for the selection of representative genotypes in future radiation-resistance breeding programs.

**Table 2:** Regression equations and LD_50_ (Gy) values of nine genotypes (G1–G9) in response to gamma irradiation. The regression equations represent the relationship between irradiation dose (x) and a given biological response (y). LD_50_ values were calculated based on the regression curves. “/” indicates that the LD_50_ value could not be determined due to the positive slope of the regression line.

Genotypes	Equation of regression	LD_50_ (Gy)
G1	y = −0.15x + 52.19	93.77
G2	y = −0.44x + 41.67	29.36
G3	y = −0.15x + 39.89	99
G4	y = −0.80x + 66.76	46.73
G5	y = −0.38x + 44.97	75.58
G6	y = −0.34x + 53.89	84.97
G7	y = −0.19x + 14.20	71.45
G8	y = 0.02x + 50.44	/
G9	y = −0.14x + 16.35	54.29

### Root and shoot growth of *Leymus chinensis* under irradiation and salt stress

**Figure 3:**
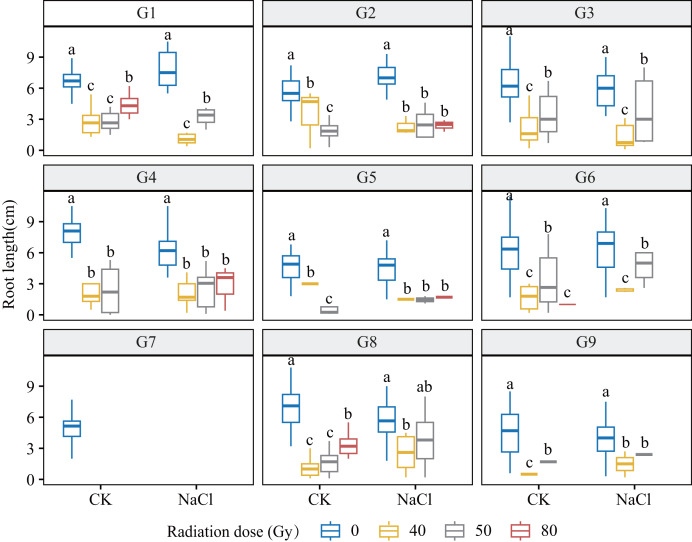
Root length (RL) of nine *L. chinensis* genotypes (G1–G9) under different carbon ion beam irradiation doses (0, 40, 50, and 80 Gy) and salinity treatments. Boxplots display the distribution of root length (cm) across radiation and salinity conditions. Different lowercase letters indicate statistically significant differences among radiation dose groups within the same salinity treatment.

**Figure 4:**
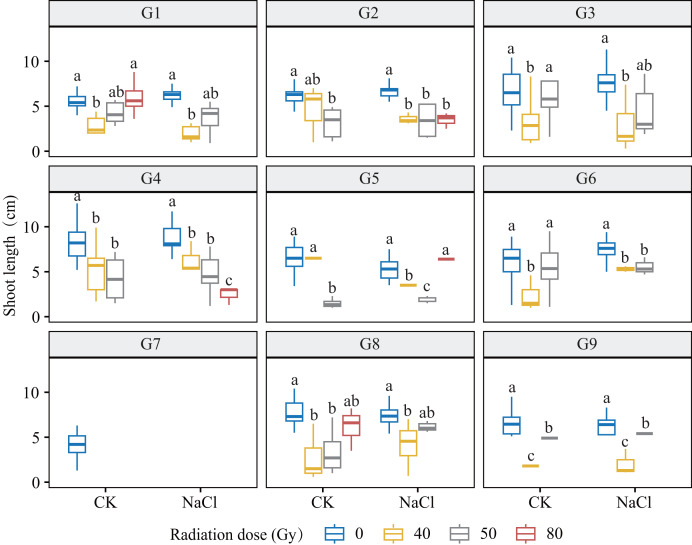
Shoot length (SL) of nine *L. chinensis* genotypes (G1–G9) in response to carbon ion beam irradiation at four dose levels (0, 40, 50, and 80 Gy) under control and salt-stress conditions. Each boxplot represents variation in shoot length (cm) under combined treatment conditions. Different lowercase letters represent significant differences between radiation doses within each salinity group.

Heavy-ion irradiation markedly suppressed root and shoot growth in *L. chinensis*, with inhibition intensifying as dose increased (Figs. 3 and 4; Table 1). Root elongation was especially impaired at 80 Gy, where most genotypes showed near-complete arrest (*p* < 0.01).

Genotypes G3 and G6 were the most sensitive: at 80 Gy, no measurable roots developed, indicating severe meristem disruption and a radiation-arrest phenotype. Conversely, G8 maintained normal germination and emergence across doses yet still exhibited a 67.3–77.1% reduction in root length at 80 Gy (*p* < 0.001), revealing latent post-germination sensitivity. Likewise, G5 displayed pronounced susceptibility; primary roots were drastically shortened at 80 Gy (*p* < 0.001) and normal root architecture failed to form, consistent with severe radiation damage. Shoot growth also displayed genotype-specific patterns. G4 and G5 showed marked inhibition under salt stress and high radiation, with shoot length nearly halved at 80 Gy. By contrast, G1 and G3 maintained relatively stable elongation at low-to-moderate doses, though still significantly reduced *vs* controls (*p* < 0.05). G9 proved highly salt-sensitive, with severe suppression even at 40 Gy under NaCl stress. Notably, G8 again exhibited relative resilience, sustaining moderate shoot growth across treatments in line with its superior germination.

### Genotype clustering and integrated trait variation *via* PCA

**Figure 5:**
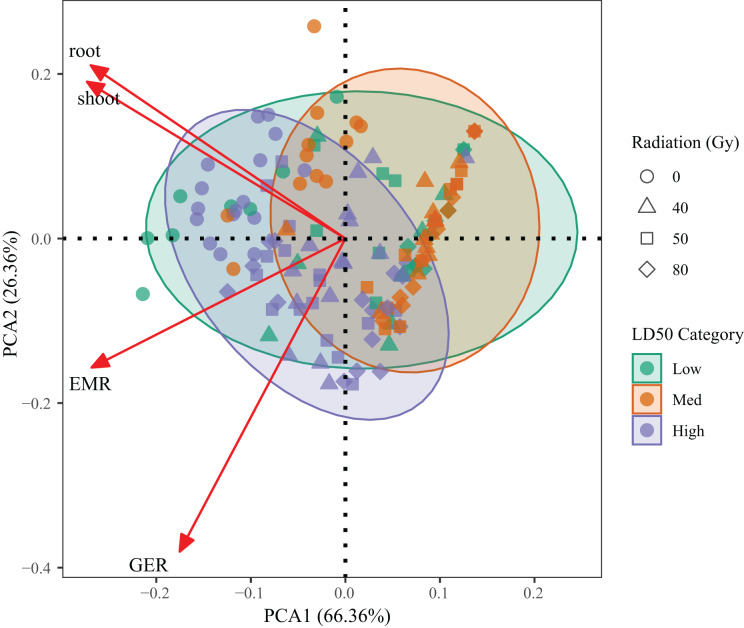
Principal component analysis (PCA) based on germination percentage, emergence percentage, root length and shoot length of *L. chinensis* under different radiation doses. Each point represents a genotype × dose combination. Colors indicate LD_50_-based resistance classes.

PCA was performed on four key traits—germination percentage, emergence percentage, root length, and shoot length—to evaluate integrated genotype responses across radiation treatments (Fig. 5). The first two components explained 92.72% of the total variance (PC1 = 66.36%, PC2 = 26.36%), indicating that the model effectively captured the dominant variation among treatments.

PC1 was mainly associated with germination and emergence performance, while PC2 reflected root and shoot growth. High-resistance genotypes (G1, G3, G6) clustered on the positive side of PC1, corresponding to superior early-stage vigor. In contrast, low-resistance genotypes (G2, G4) were located in the lower-left quadrant, indicating suppressed growth across all indicators. Medium-resistance genotypes (G5, G7, G9) occupied intermediate positions, reflecting partial tolerance.

A clear trend across radiation doses was also observed, with most 80 Gy samples diverging from the high-performance quadrant, confirming broad inhibitory effects at high doses. These results validate the LD_50_-based resistance classification and provide a robust multivariate framework for assessing genotype performance under radiation stress.

## Discussion

This study evaluated the genotype-specific phenotypic responses of *L. chinensis* to HIBI during seed germination and early seedling development, particularly under salt stress conditions. Significant interactive effects were observed among radiation dose, salt stress, and genotype. Germination percentage, seedling emergence rate, root length, and shoot height exhibited clear dose-dependent responses and substantial physiological differentiation, reflecting the influence of genetic background.

Low-dose irradiation (≤40 Gy) significantly enhanced germination in specific genotypes (*e.g*., G3, G8, G9), even under salt stress, suggesting a potential adaptive advantage under combined abiotic stress. Such enhancement is often attributed to hormetic responses, in which moderate oxidative stress induces antioxidant defenses, stabilizing membranes and improving water uptake, thereby mitigating NaCl-induced ion toxicity (Abogadallah, Serag & Quick, 2010; Li et al., 2020; Wang et al., 2018a, 2022; Yin et al., 2023). Both radiation and salt stress are known to trigger ROS accumulation, and their interaction may result in either synergistic damage or cross-protection depending on stress intensity. Consistently, G9 showed a 65.1% increase in germination even under high-dose irradiation, indicating that ROS-mediated cross-talk between radiation and salt stress signaling may contribute to its tolerance. Comparable regulatory mechanisms have been identified in other crops: in soybeans, salt tolerance was associated with cuticle reinforcement, linolenic acid metabolism, and DNA methylation (Fu et al., 2024), while in alfalfa, enhanced proline synthesis together with the activation of antioxidant enzyme genes (*e.g*., *APX* and *Cu/Zn-SOD*) synergistically improved salt tolerance (Guo et al., 2022). These parallels suggest that *L. chinensis* may rely on similar ROS-centered regulatory networks to cope with combined stresses.

In contrast, high-dose irradiation (≥50 Gy) exerted strong inhibitory effects on most genotypes, particularly G4 and G2, which showed drastic reductions in germination percentage and seedling emergence. G7 was especially sensitive in terms of root length, with its survival rate dropping below 10% under 80 Gy. These effects are likely due to cumulative DNA double-strand breaks, cell cycle arrest, and the initiation of programmed cell death (Ling et al., 2024). When combined with salt stress, radiation intensified growth inhibition, likely through additive or synergistic effects on ROS overload, ion toxicity, and osmotic imbalance, thereby overwhelming cellular defense systems (Jing et al., 2024). Notably, unlike other forage species such as *Medicago sativa* or *Festuca arundinacea*, which can partially counteract stress via antioxidant activation under low-dose irradiation (Amirikhah et al., 2021; Kim et al., 2019; Putra & Prasetya, 2024; Rejili et al., 2008; Zhang et al., 2024) *L. chinensis*, as a perennial species, may exhibit higher sensitivity to high-dose radiation owing to lower meristematic repair capacity and a longer cell cycle duration (Caplin, Halliday & Willey, 2020; Pan et al., 2018). This highlights the importance of considering the interactive mechanisms of radiation and salinity when evaluating stress tolerance in perennial forage grasses.

The observed variation in stress responses among genotypes highlights the decisive role of genetic background (Rezk et al., 2019; Yang et al., 2019). Genotype G8 showed stable germination and emergence across treatments, suggesting innate tolerance to both radiation and salt stress, potentially linked to efficient ROS detoxification and DNA repair pathways such as ATM and RAD51 (Jing et al., 2019; Šamanić et al., 2016); G5 maintained stable germination but showed reduced growth, indicating stage-specific vulnerability when dividing tissues are challenged by combined stresses. By contrast, G7 was highly sensitive, possibly reflecting genomic instability and weaker ROS-scavenging capacity. The large, highly heterozygous genome of *L. chinensis* may also influence its ability to regulate stress-responsive mutations (Ahmed & Hou, 2022; Hunter, McCarty & Koch, 2023; Li et al., 2023).

From an applied perspective, this study estimated LD_50_ values based on seedling emergence and used PCA to integrate multiple traits, establishing a resistance classification framework. PCA results revealed distinct distributions of high-, medium-, and low-resistance genotypes along the principal component axes, underscoring strong trait co-variation and the feasibility of early-stage phenotypic screening. G3 and G9 were particularly notable, exhibiting superior performance across traits, and should be prioritized as candidate lines for mutant selection.

It is worth noting that the application of carbon ion beam mutagenesis combined with screening in perennial forage grasses still faces challenges. Although this study provides preliminary evidence, it has three limitations: (1) the limited number of genotypes tested restricts the representation of *L. chinensis* diversity; (2) the absence of physiological and molecular data (*e.g*., ROS levels, antioxidant enzyme activity, ion fluxes, or DNA repair gene expression) constrains deeper mechanistic understanding of radiation–salt interactions; (3) all experiments were conducted indoors, and the field performance of potential mutants under saline-alkali conditions remains to be tested.

In summary, HIBI elicited strong dose-dependent effects on germination and early growth in *L. chinensis*, with substantial genotype-dependent variation. The findings suggest that the interaction between radiation and salt stress may involve a dynamic balance among ROS priming and overload, DNA repair capacity, and ion homeostasis. Genotypes such as G3 and G9 demonstrated promising tolerance to both stresses, making them valuable candidates for developing salt-tolerant germplasm. This study provides a practical framework for phenotypic screening and mutant selection in perennial forage breeding. Future research integrating multi-omics approaches will be crucial to unravel the regulatory networks underlying radiation–salt interactions and accelerate the development of elite *L. chinensis* cultivars for saline-alkali land restoration and sustainable grassland agriculture.

## Conclusion

This study demonstrated that carbon ion beam irradiation induces significant, dose-dependent effects on seed germination and early seedling growth in *L. chinensis*, with pronounced genotype-specific variation under both saline and non-saline conditions. Certain genotypes (*e.g*., G3, G6) responded positively to low-dose exposure, whereas others (*e.g*., G2, G4) were markedly sensitive to increasing doses. Salt stress further amplified these differences, with G3 and G9 maintaining or improving performance even under high-dose conditions, indicating strong radiation tolerance and salinity resilience. Based on LD_50_ values and PCA analysis, genotypes were effectively grouped into high, medium, and low resistance categories. The integrative analysis revealed consistent covariation among multiple traits, providing a reliable framework for identifying germplasm with enhanced tolerance profiles. These findings demonstrate the potential of carbon ion irradiation to generate beneficial variation in perennial forage species and highlight the critical role of genotype–dose interactions in shaping adaptive responses.

## Supplemental Information

10.7717/peerj.20510/supp-1 Supplemental Information 1 Raw data for germination percentage and seedling emergence percentage. Genotype-level measurements across all radiation dose and salt stress treatments. Data were used for LD_50_ calculation and resistance classification.

10.7717/peerj.20510/supp-2 Supplemental Information 2 Raw data for root length. Individual root length measurements of *Leymus chinensis* seedlings under different genotype × dose × stress combinations, used for phenotypic response analysis.

10.7717/peerj.20510/supp-3 Supplemental Information 3 Raw data for seedling (shoot) length. Shoot length data for each treatment group and genotype. The values contributed to PCA and the evaluation of early-stage growth performance.

10.7717/peerj.20510/supp-4 Supplemental Information 4 Key morphological characteristics of the nine *Leymus chinensis* genotypes used in this study. Origin and key morphological characteristics of the *Leymus chinensis* materials, including officially released cultivars, breeding lines under regional trials, and wild accessions collected from representative grassland habitats.

## Additional Information and Declarations

### Data Availability

The following information was supplied regarding data availability:

The raw data used in the analyses are available in the Supplemental File.
