# Supplementary material for: Heavy ion beam-induced variation in salt tolerance among Leymus chinensis genotypes during seed germination
Source: PeerJ. 2025 Dec 17;13:e20510. doi: 10.7717/peerj.20510 (PMC12717846; doi:10.7717/peerj.20510)
Supplement: Supplemental Information 4 — Origin and key morphological characteristics of the Leymus chinensis materials, including officially released cultivars, breeding lines under regional trials, and wild accessions collected from representative grassland habitats. [file peerj-13-20510-s004.docx]

**Table S1. Key morphological characteristics of the nine *Leymus chinensis* genotypes used in this study.**

| **Genotypes** | **Type** | **Origin** | **Key morphological traits** | **TWS(g)** |
| --- | --- | --- | --- | --- |
| **G1** | Cultivar (“Dongdi 4”) | Developed by NIGA, CAS | Tall plants with large leaves | 2.82 |
| **G2** | Breeding line (Songnen 1) | Developed by NIGA, CAS | High seed yield and large biomass | 2.80 |
| **G3** | Cultivar candidate | Developed by NIGA, CAS | Large seed yield and high fruit setting rate | 2.84 |
| **G4** | Cultivar (“Dongdi 6”) | Developed by NIGA, CAS | Strong resistance to adversity and high germination rate | 2.77 |
| **G5** | Breeding line (Songnen 4) | Developed by NIGA, CAS | High seed quality and biomass | 2.85 |
| **G6** | Wild accession | Songnen Grassland, Jilin | Strong drought resistance | 2.42 |
| **G7** | Cultivar (“Dongdi 5”) | Developed by NIGA, CAS | Many tillers and large biomass | 2.44 |
| **G8** | Cultivar candidate | Developed by NIGA, CAS | Large and long ears | 2.81 |
| **G9** | Cultivar candidate | Developed by NIGA, CAS | Many tillers and large biomass | 2.60 |

Abbreviations: TSW = Thousand-seed weight (g); CAS = Chinese Academy of Sciences; NIGA = Northeast Institute of Geography and Agroecology.
